# Child nutrition to new stage in China: evidence from a series of national surveys, 1985–2015

**DOI:** 10.1186/s12889-019-6699-z

**Published:** 2019-04-11

**Authors:** Xin-Nan Zong, Hui Li, Ya-Qin Zhang, Hua-Hong Wu

**Affiliations:** Department of Growth and Development, Capital Institute of Pediatrics, Beijing, 100020 China

**Keywords:** Stunting, Underweight, Wasted, Overweight, Obesity, Children

## Abstract

**Background:**

Both child under- and over-nutrition are major global public health challenges. We aimed to examine thirty-year trends in physical growth, under- and over-nutrition in Chinese urban and suburban children between 1985 and 2015, and discuss implications for child health programmes.

**Methods:**

A total of 610,785 urban and suburban children from birth to 7 years of age were collected from a series of large-scale national surveys in China. Height, weight and body mass index (BMI) Z-scores and prevalence of stunting, underweight, wasted and possible risk of overweight, overweight and obesity were calculated according to the World Health Organization (WHO) 2006 growth standards. The trends in the prevalence were tested across different survey years by Cochran-Armitage trend test.

**Results:**

Rapid secular growth trend was observed in China over the past 30 years, but the trend showed a slowing sign in urban children in recent 10 years. The growth level of Chinese urban and suburban children surpassed the WHO 2006 growth standards in 2015. Between 1985 and 2015 the stunting, underweight and wasted prevalence decreased from 12.21, 4.44, 1.68 to 0.97%, 0.59, 0.87% for children under 5 years and from 12.69, 10.02, 3.41 to 0.42%, 0.67, 2.17% for children aged 5- < 7 respectively; the possible risk of overweight prevalence increased from 6.51 to 12.57%, overweight from 0.70 to 3.48% and obesity from 0.17 to 0.86% for children aged 2- < 7 and the increasing rates of overweight and obesity prevalence in suburban children first outnumbered urban children in recent 10 years. The overweight prevalence overtook the wasted or underweight in children aged 2- < 7 in 2005 and onward.

**Conclusion:**

Slowing secular height trend and overweight prevalence overtaking the wasted or underweight suggested child nutrition and health strategies should adjust swiftly and deliberately from primarily reducing under-nutrition prevalence to controlling rapid weight gain and promoting integrated early development.

## Background

Nutritional status of children was one of the most important global public health issues. In 1975, China launched the National Survey on Physical Growth and Development of Children (NSPGDC) in nine cities in China [1, 2]. Thereafter, the NSPGDC was repeatedly undertaken using the same methods in the same nine cities for the continuity and comparability of the survey every 10 years apart [3–5]. The series of the NSPGDC was the largest nationally representative sample of children under 7 years in China. Data from each survey year were used as the growth reference for Chinese children and the scientific evidence for developing child nutrition and health policies in China [6, 7]. The first four NSPGDC showed rapid positive secular trend in height and weight in both urban and suburban children from 1975 to 2005 [8], but the 5th NSPGDC conducted in 2015 displayed a new sign of slowing growth trend in urban children [9]. Now is a great opportunity to comprehensively re-evaluate growth and nutrition among Chinese children. Early childhood nutrition is the foundation of sustainable development for all. Both child under- and over-nutrition are major global public health challenges [10, 11], progress and challenges from China may have useful implications for many parts of the world.

Since 1978, an evolution of the Chinese economic policy from country’s planned economy to free market system has increased average household income and personal expenditure on food and health. Socioeconomic development after the 1978 economic reforms has been accompanied by continuous release of genetic growth potential [12, 13]. Since early 1990s, the Chinese diet pattern has changed towards higher fat and calories and lower dietary fibre [14] which has increased the risk of overweight and obesity among children [15]. Many parts of the world are undergoing transition from low- to middle-income and economic conditions in these countries or regions are similar to China. Rapid transition from under-nutrition to over-nutrition was documented in some low- and middle-income countries [16, 17]. Therefore, the implications from China may be helpful to further develop child nutrition and health strategies for many parts of the world.

The objectives of this study were to examine thirty-year trends in physical growth, under- and over-nutrition in urban and suburban children in China between and 2015 and discuss implications for child health programmes. In addition, we also examined the association between secular height trend and socioeconomic status.

## Methods

### Study subjects

**Fig. 1:**
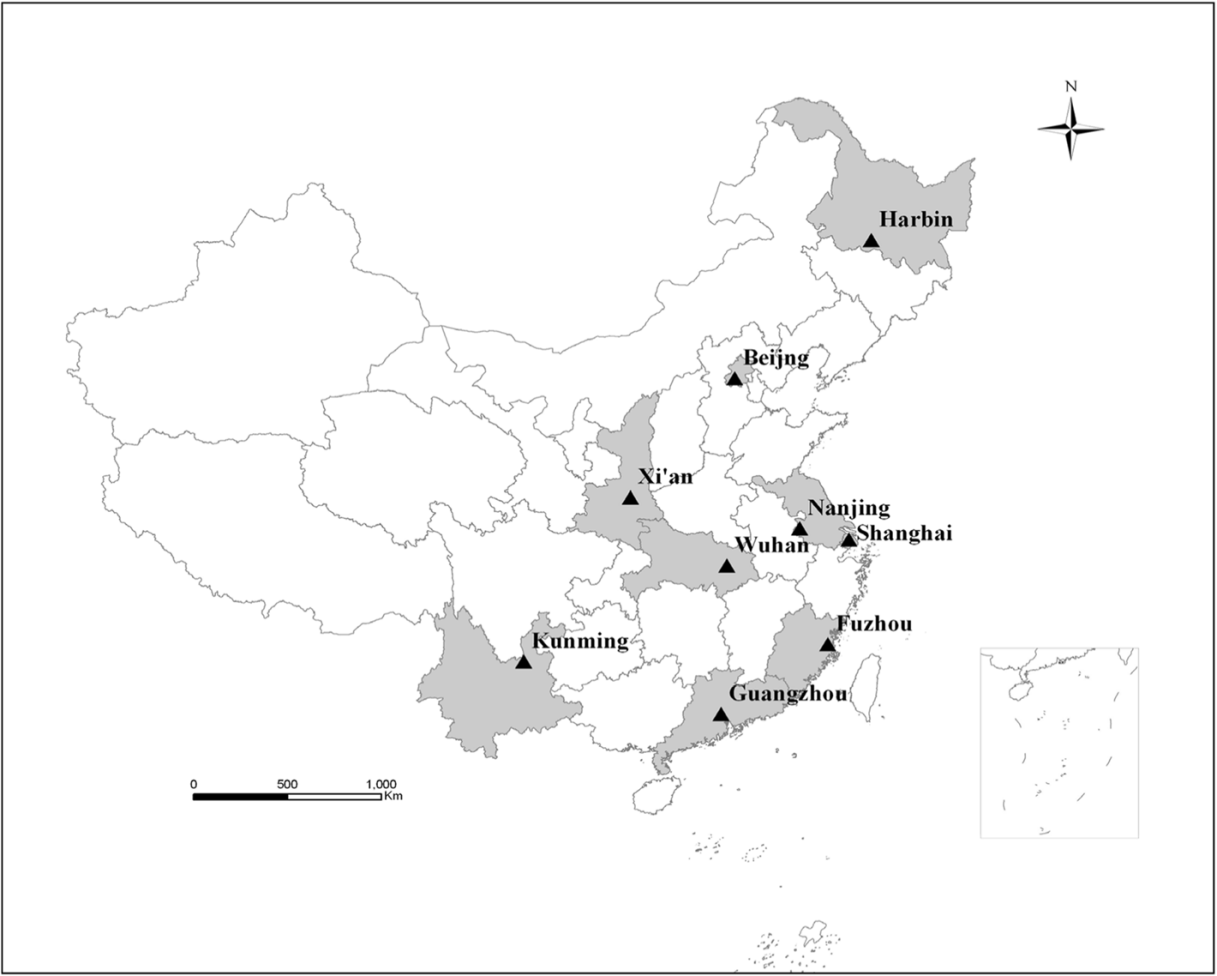
Geographical distribution of the nine surveyed cities (Shaded their corresponding provinces) in China

Children from birth to 7 years were obtained from the four rounds of the NSPGDC repeatedly carried out in nine cities in China in 1985, 1995, 2005 and 2015. Of these cities, Beijing and Shanghai are province-level municipalities directly under the Central Government, and the other seven cities are provincial capital cities, including Harbin (Heilongjiang’s provincial capital), Xi’an (Shaanxi), Nanjing (Jiangsu), Wuhan (Hubei), Guangzhou (Guangdong), Fuzhou (Fujian), and Kunming (Yunnan) (Fig. 1). Healthy children under 7 years old are divided into 22 age groups: newborn to 3 days, monthly for 1- < 6 months, bi-monthly for 6- < 12 months, tri-monthly for 12- < 24 months, half-yearly for 2- < 6 years, and yearly for 6- < 7 years. Each sex-age subgroup consists of about 200 children in urban/suburban areas of each of nine cities. Urban children are defined as permanently living in urban area in the surveyed city, or these children moved into the surveyed city from other large cities and lived time more than two-thirds of their own ages; and suburban children as either one or both parents are farmers and they are brought up in the suburban area (surrounding the surveyed city). The NSPGDC used stratified cluster sampling method according to urban/suburban areas and administrative districts in each of nine cities. Children under 3 years old come from the community (as a minimum cluster unit) and children aged 3- < 7 from the kindergartens (as a minimum cluster unit) in each selected administrative. Exclusion criteria are temporary residents, history of premature birth, birth weight below 2.5 kg, twins or multiple births, acute illness within a month, chronic illness, obviously malnourished and physically handicap.

A total of 610,785 eligible children from birth to 7 years of age were collected from four rounds of the NSPGDC between 1985 and 2015 (Table 1). Four subpopulations of urban and suburban boys and girls were almost equal sample sizes in each survey year.

**Table 1:** Basic characteristics of urban and suburban subpopulations in the NSPGDC at each survey year, 1985–2015

	Urban				Suburban			
	1985	1995	2005	2015	1985	1995	2005	2015
Sample sizes	79,194	79,154	69,760	83,628	73,680	78,208	69,015	78,146
Feeding patterns within 6 months (%)								
Exclusive or predominant breastfeeding	33.6	53.5	32.8	52.9	60.2	63.1	42.5	53.8
Partial breastfeeding	43.4	32.7	47.1	34.2	33.1	25.6	41.9	31.0
Formula feeding	23.0	13.8	20.1	12.9	6.7	11.3	15.6	15.2
College and above (%)								
Father	–	29.4	49.3	69.6	–	2.9	10.8	37.2
Mother	–	21.9	43.7	67.9	–	1.4	8.1	35.8
Annual family income ≥50 thousand Yuan (%) ^a^	–	–	35.8	78.2	–	–	9.6	61.5

^a^1 Yuan is approximately equal to 0.16 US dollar

### Organization, measurements and quality control

Under the direction and supervision of the Beijing Steering Committee, the nine coordinating study subgroups set up in each of nine cities worked simultaneously following a uniform plan, organizing and training local survey teams, carrying out the measurements and checking cards. The survey started in May and finished in October of the same year. Principal investigator, other two key investigators and leader of the local Health Commission in charge of the investigation in each city participated in national training course held by the Beijing Steering Committee who organized and trained local doctors, technicians or nurses following the technical requirements of national training course. All the field investigators participated in rigorous training and passed an examination before starting the survey. The Beijing Steering Committee supervised the implementation of the project in each of nine cities and delegated two or three senior technicians to each surveyed city. Unified measuring tools/instruments were equipped for each field sites of each of nine cities.

Weight and height (not in shoes) were measured using unified measuring tools/instruments in a standardized way by specially trained technicians or nurses. Weight was measured with lever scale to the nearest 0.01 kg with children wearing the lightest vest, shorts or underwear. Height was measured supine with horizontal metal infantometer for children under 3 years, while metal column height and sitting height measuring device was applied to children aged 3- < 7. All height measurements were recorded to the nearest 0.1 cm. Errors of weight and height were not more than 0.05 kg or 0.5 cm between two repeated measurements. All the physical measurements were carried out at least 1 h after a meal between approximately 8 am and 4 pm. Feeding patterns of infants, education background of parents and family income were collected simultaneously by means of face-to-face visit to children’s parents.

### Socioeconomic data

Captured as the proxy of socioeconomic data, gross domestic product (GDP) per capita and infant mortality rate (IMR) in China from 1985 to 2015 were obtained from the 2016 World Development Indicators from the World Bank website (https://datacatalog.worldbank.org/dataset/world-development-indicators).

### Statistical analysis

BMI is calculated as body weight (kg) divided by the square of height (m). We generated Z-scores of height, weight and BMI using the Cole’s LMS method [18] and prevalence of stunting (below − 2 standard deviations (SD) from median for length/height-for-age), underweight (below − 2 SD from median for weight-for-age), wasted (below − 2 SD from median for weight-for-length/height for children under 5 years and for BMI-for-age for children aged 5- < 7), possible risk of overweight (above + 1 SD from median for weight-for-length/height for children under 5 and for BMI-for-age for children aged 5- < 7), overweight (above + 2 SD from median) and obesity (above + 3 SD from median) based on the WHO 2006 growth standards for children under 5 [19] and the WHO 2007 growth reference for children aged 5- < 19 [20]. We calculated Pearson’s correlation coefficients between mean height Z-score and GDP per capita and IMR. Thirty-year trends in the prevalence of stunting, underweight, wasted and possible risk of overweight, overweight and obesity were tested across different survey years by Cochran-Armitage trend test. Data analysis was performed in SAS v9.4 (SAS Institute, Cary, North Carolina).

## Results

### Basic characteristics

The 1985, 1995, 2005 and 2015 NSPGDC were composed of 152,874, 157,362, 138,775 and 161,774 children from birth to 7 years respectively, with both boys and girls consisting of approximately equal numbers from urban and suburban areas of nine cities. The percentage of exclusive or predominant breastfeeding within 6 months were 33.6% for urban children and 60.2% for suburban children in 1985, 53.5 and 63.1% in 1995, 32.8 and 42.5% in 2005 and 52.9 and 53.8% in 2015 (Table 1). The percentages of fathers and mothers with college and above presented a steadily increasing from 29.4 and 21.9% in urban subpopulation and 2.9 and 1.4% in the suburban in 1995 to 69.6 and 67.9%, and 37.2 and 35.8% in 2015 respectively. Annual family income more than 50 thousand Yuan accounted for 35.8% in urban subpopulation and 9.6% in suburban subpopulation in 2005 and 78.2 and 61.5% in 2015 respectively.

### Height and stunting

#### Height Z-scores

**Fig. 2:**
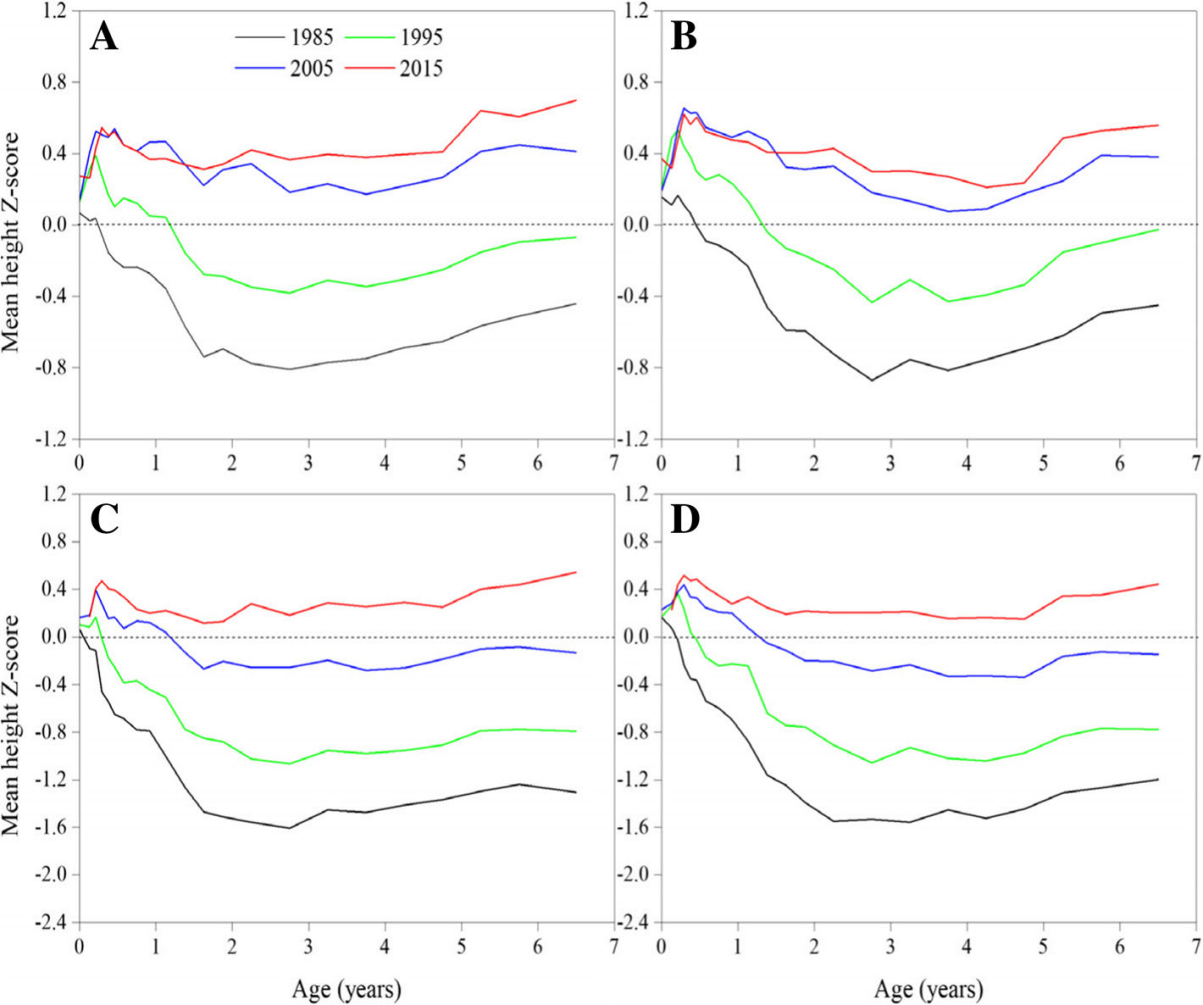
Thirty-year trends in mean height Z-score in children under 7 years by sex and area, 1985–2015. (**a**: urban boys, **b**: urban girls, **c**: suburban boys, **d**: suburban girls)

Mean height Z-scores showed rapid positive trends for urban children between 1985 and 2005 and for suburban children between 1985 and 2015 (Fig. 2). The changing increments of height Z-scores trends presented a narrowing sign for urban children in recent 10 years, and in particular the height Z-scores almost did not increase for infants and toddlers. Generally, the growth performance of height for urban children surpassed the WHO 2006 growth standards in 2005 and onward and for suburban children in 2015.

#### Associations of height with GDP and IMR

**Fig. 3:**
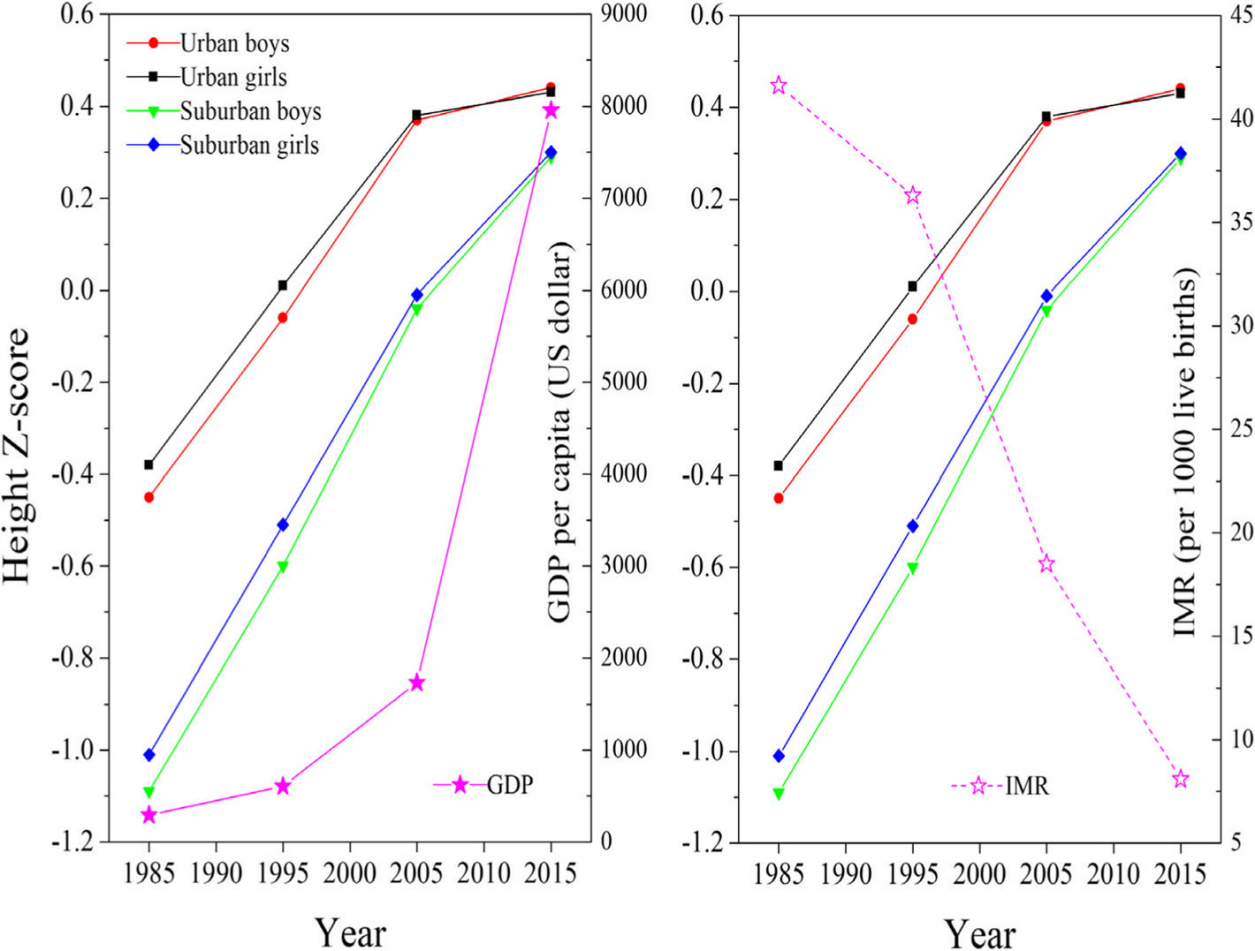
Thirty-year trends in mean height Z-scores in children under 7 years, GDP per capita and IMR in China, 1985–2015

Figure 3 illustrated a tendency of slowing changing for mean height Z-score trends for urban children in recent 10 years, and rapid growth for GDP per capita and sharp decline for IMR over the past 30 years. Height Z-scores were strongly positively associated with GDP per capita, with being stronger correlation between 1985 and 2005 than between 1985 and 2015 (Table 2). Further, the correlation coefficients with GDP per capita seemed to be larger in suburban than urban subpopulations between 1985 and 2015. On the contrary, however, height Z-scores showed strong negative correlation with IMR adjusted by GDP per capita.

**Table 2:** Pearson correlation coefficients (r) between mean height Z-scores of children under 7 years and GDP per capita and IMR

	r__1_	p__1_	r__2_	p__2_
Height Z-score with GDP per capita				
Urban boys	0.96	0.18	0.71	0.29
Urban girls	0.95	0.21	0.69	0.31
Suburban boys	0.96	0.18	0.82	0.18
Suburban girls	0.95	0.20	0.81	0.19
Height Z-score with IMR ^a^				
Urban boys	–	–	−0.95	0.20
Urban girls	–	–	−0.93	0.23
Suburban boys	–	–	−0.94	0.20
Suburban girls	–	–	−0.93	0.23

r__1_ Association between 1985 and 2005; r__2_ Association between 1985 and 2015; p__1_ *P*-value for r__1;_ p__2_ P-value for r__2_

^a^Adjusted by GDP per capita

#### Stunting

**Fig. 4:**
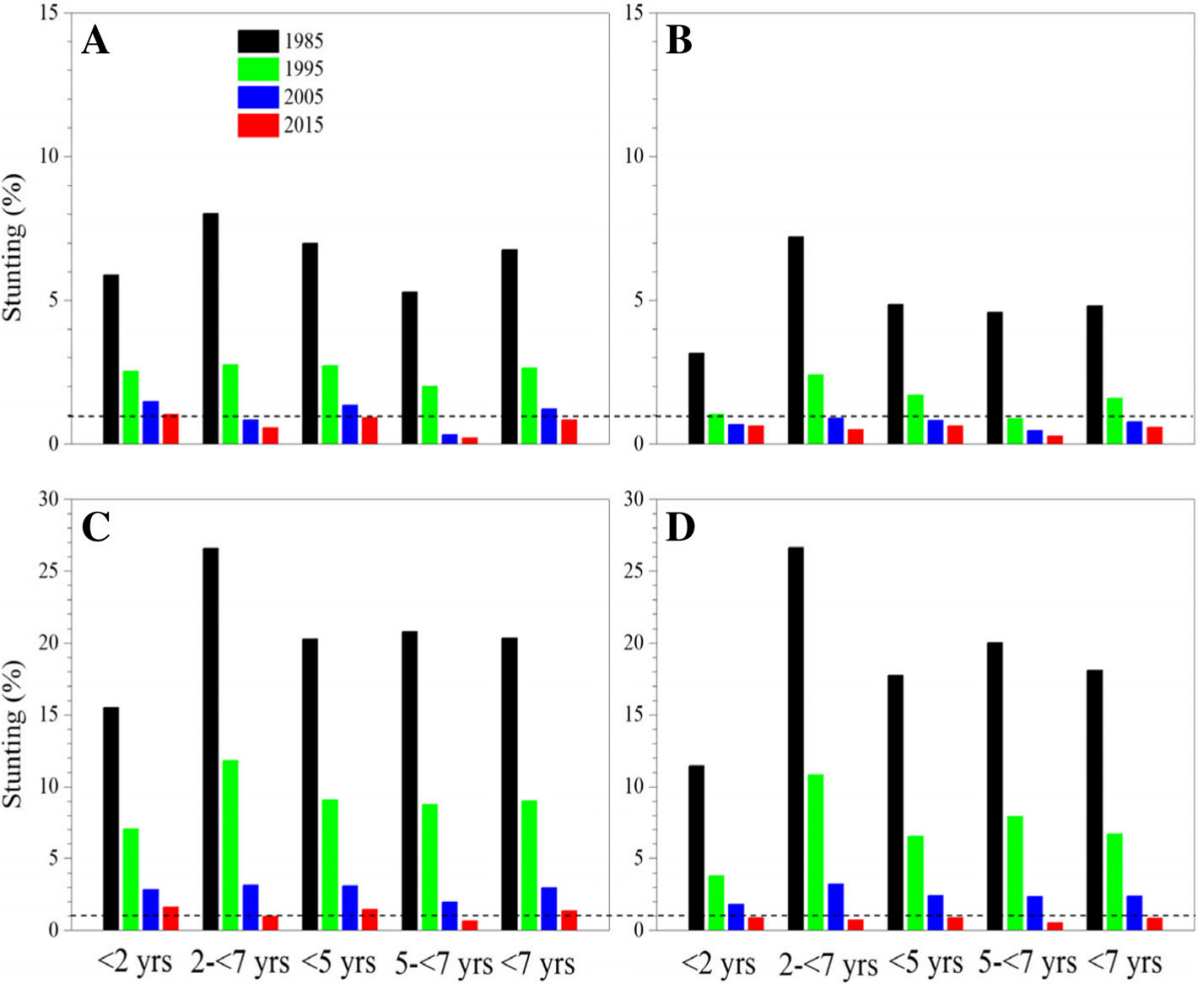
Thirty-year trends in stunting prevalence in children by age, sex and area, 1985–2015. (**a**: urban boys, **b**: urban girls, **c**: suburban boys, **d**: suburban girls)

The stunting prevalence declined dramatically from 12.21 to 0.97% for children under 5 years and from 12.69 to 0.42% for children aged 5- < 7 from 1985 to 2015 (Table 3). By 2015, the prevalence declined to 0.93% for urban boys, 0.63% for urban girls, 1.47% for suburban boys and 0.88% for suburban girls under 5 years, and to 0.21, 0.28, 0.67 and 0.53% for children aged 5- < 7 respectively (Fig. 4).

**Table 3:** Thirty-year trends in underweight, stunting, wasted, possible risk of overweight, overweight and obesity prevalence in children by age, 1985–2015

	1985	1995	2005	2015	χ^2^	P for trend
< 2 yrs						
Underweight	3.23	1.09	0.72	0.57	2952.11	< 0.0001
Stunting	8.73	3.59	1.69	1.03	8910.29	< 0.0001
Wasted	1.88	1.28	1.08	0.85	416.09	< 0.0001
Possible risk of overweight ^a^	19.13	23.25	26.13	25.82	1525.68	< 0.0001
Overweight ^b^	3.58	4.42	5.53	5.60		
Obesity	0.48	0.48	0.71	0.74		
2- < 7 yrs						
Underweight	7.94	3.15	1.27	0.64	6689.55	< 0.0001
Stunting	17.12	6.95	2.04	0.70	17,068.44	< 0.0001
Wasted	1.99	1.54	1.32	1.32	123.98	< 0.0001
Possible risk of overweight ^a^	7.38	8.40	13.64	16.91	3875.34	< 0.0001
Overweight ^b^	0.87	0.81	2.32	4.34		
Obesity	0.17	0.09	0.24	0.86		
< 5 yrs						
Underweight	4.44	1.61	0.84	0.59	6695.22	< 0.0001
Stunting	12.21	4.99	1.92	0.97	21,373.95	< 0.0001
Wasted	1.68	1.20	1.01	0.87	433.97	< 0.0001
Possible risk of overweight ^a^	15.92	19.01	22.41	22.73	2563.41	< 0.0001
Overweight ^b^	2.76	3.30	4.48	4.96		
vObesity	0.40	0.36	0.57	0.72		
5- < 7 yrs						
Underweight	10.02	3.99	1.59	0.67	2920.65	< 0.0001
Stunting	12.69	4.89	1.29	0.42	4367.71	< 0.0001
Wasted	3.41	2.62	2.30	2.17	77.99	< 0.0001
Possible risk of overweight ^a^	3.49	5.42	11.87	17.94	3259.36	< 0.0001
Overweight ^b^	0.49	0.69	2.42	5.73		
Obesity	0.07	0.09	0.18	1.21		
< 7 yrs						
Underweight	5.23	1.94	0.95	0.60	9567.42	< 0.0001
Stunting	12.28	4.97	1.83	0.89	25,683.72	< 0.0001
Wasted	1.93	1.38	1.19	1.05	502.54	< 0.0001
Possible risk of overweight ^a^	14.17	17.14	20.95	22.06	3992.93	< 0.0001
Overweight ^b^	2.44	2.94	4.20	5.07		
Obesity	0.35	0.32	0.52	0.79		

^a^Including overweight and obesity; ^b^ Including obesity

### Weight and underweight

#### Weight Z-scores

**Fig. 5:**
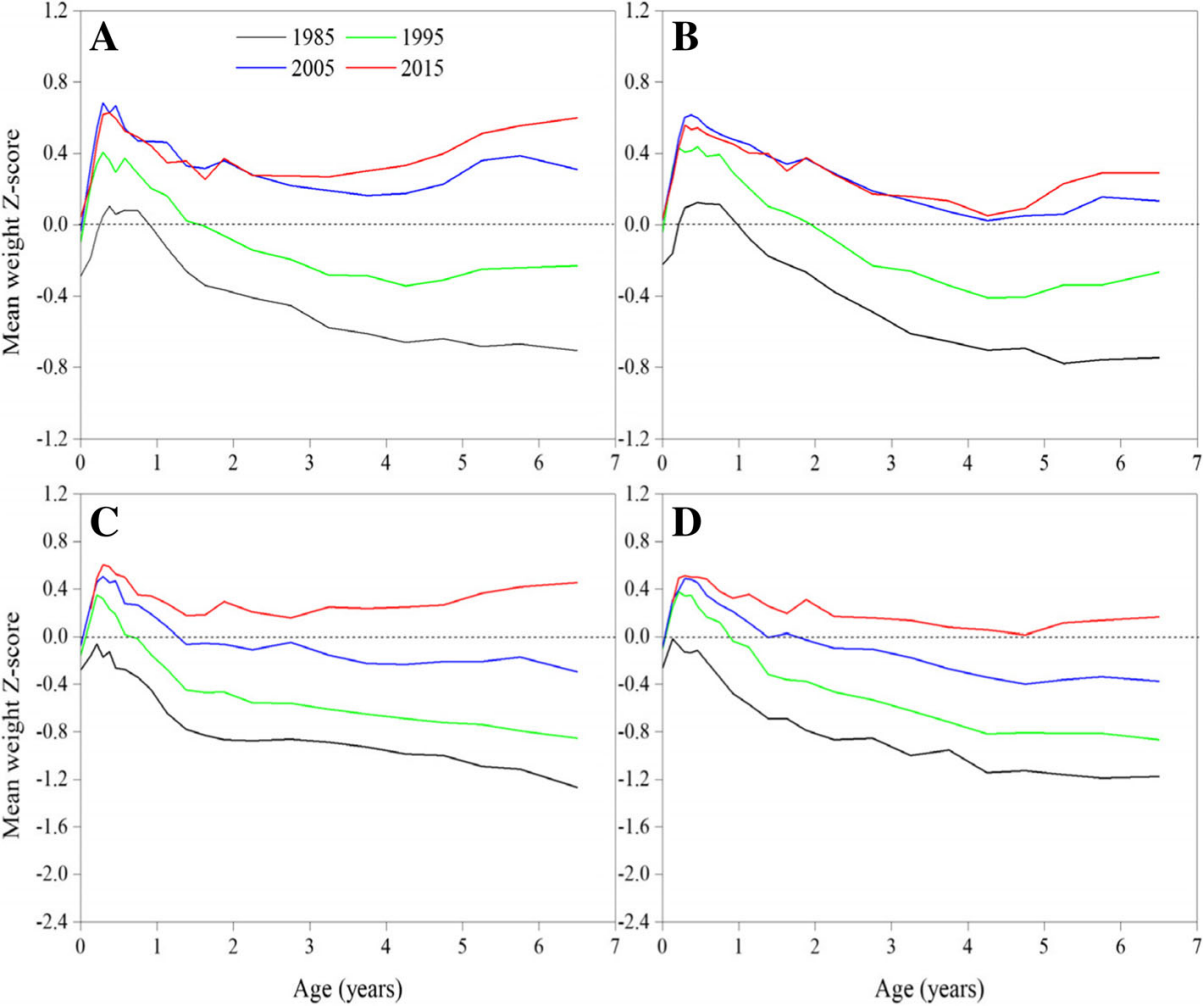
Thirty-year trends in mean weight Z-score in children under 7 years by sex and area, 1985–2015. (**a**: urban boys, **b**: urban girls, **c**: suburban boys, **d**: suburban girls)

Similar to the growth performance of height, the growth level of weight has also experienced the process that lowered the WHO 2006 growth standards before 1995, reached in 2005 and onward for urban children and surpassed in 2015 for both urban and suburban children (Fig. 5). Mean weight Z-scores trends also displayed a slowing sign in recent 10 years.

#### Underweight

**Fig. 6:**
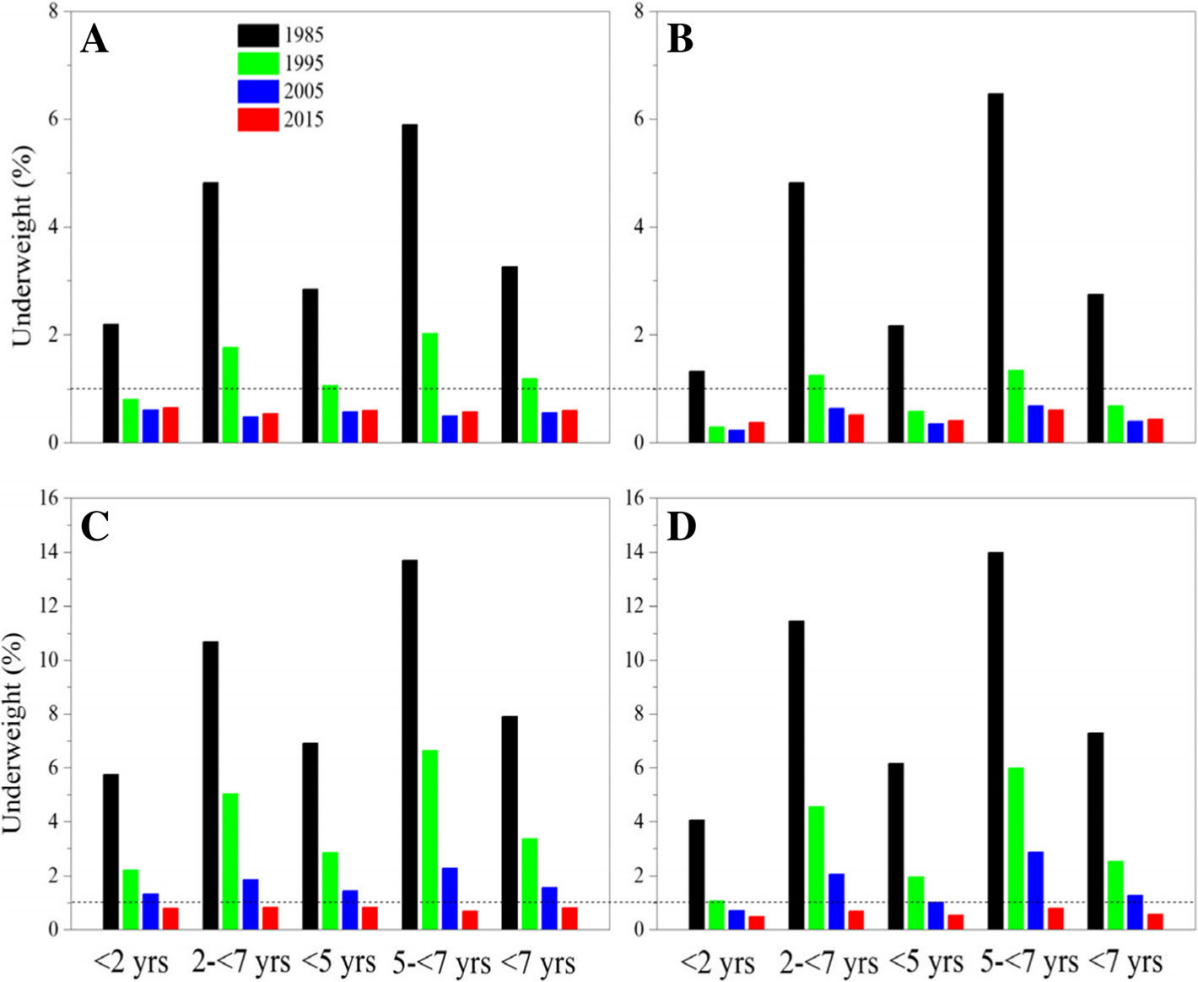
Thirty-year trends in underweight prevalence in children by age, sex and area, 1985–2015. (**a**: urban boys, **b**: urban girls, **c**: suburban boys, **d**: suburban girls)

The underweight prevalence decreased dramatically from 4.44 to 0.59% for children under 5 years and from 10.02 to 0.67% for children aged 5- < 7 from 1985 to 2015 (Table 3). The prevalence of less than 1% of level occurred in 2005 and onward for urban children and in 2015 for suburban children (Fig. 6).

### BMI and wasted and overweight

#### BMI Z-scores

**Fig. 7:**
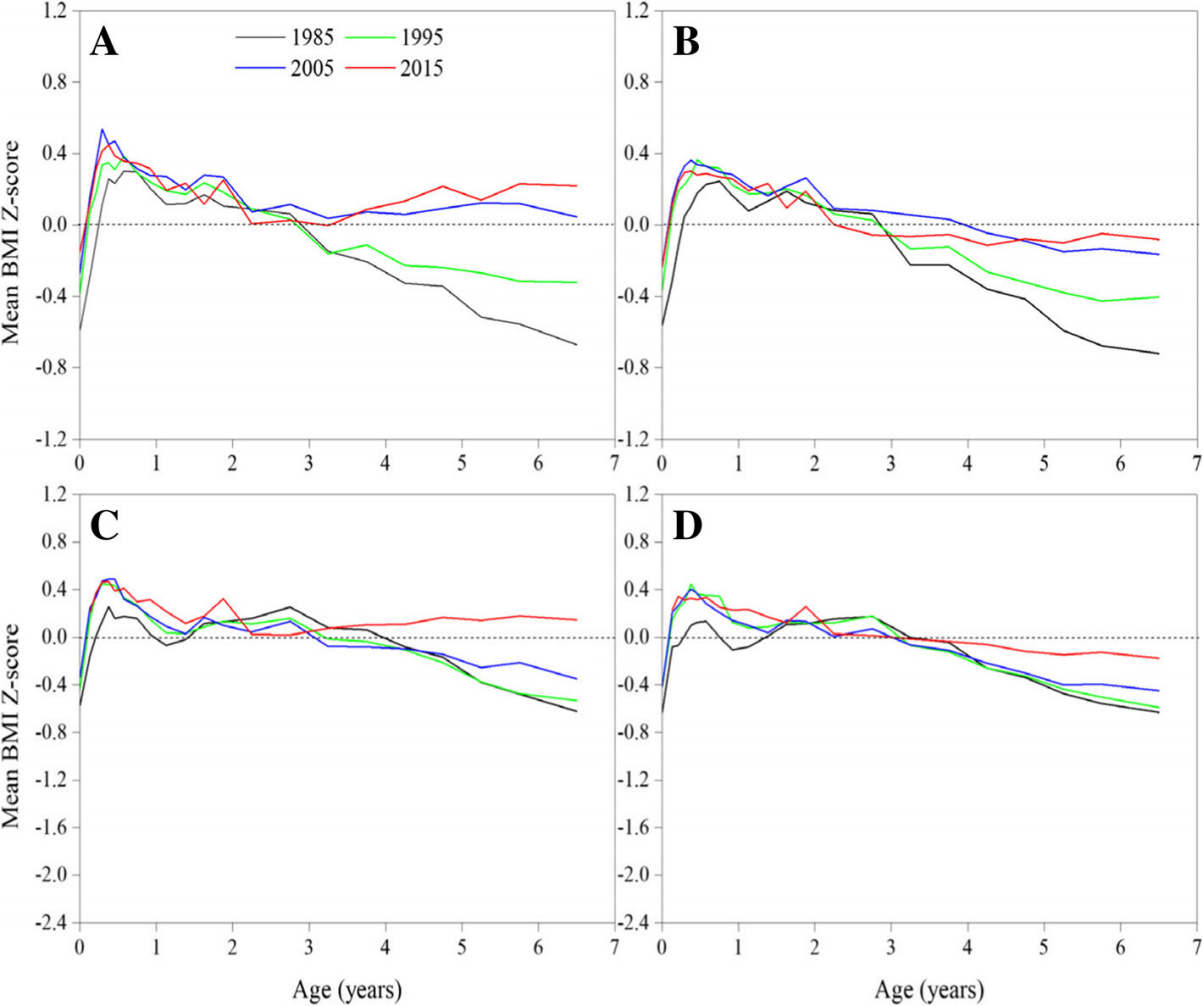
Thirty-year trends in mean BMI Z-score in children under 7 years by sex and area, 1985–2015. (**a**: urban boys, **b**: urban girls, **c**: suburban boys, **d**: suburban girls)

**Fig. 8:**
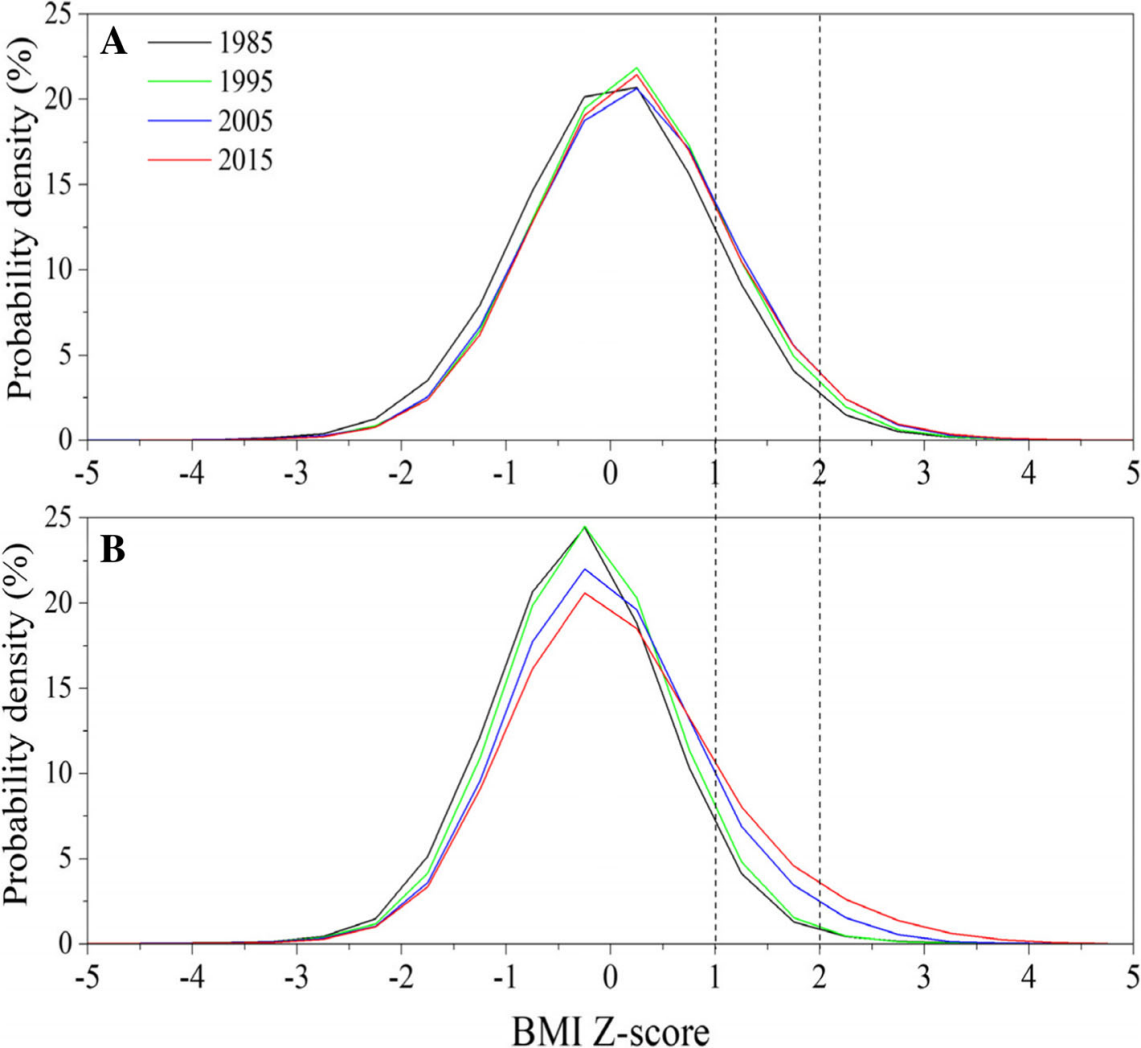
Thirty-year trends in probability density of BMI Z-scores in children by age, 1985–2015. (**a**: children under 3 years, **b**: children aged 3- < 7)

A clear positive trend in mean BMI Z-scores was observed in urban children aged 3- < 7 between 1985 and 2005 and in suburban children between 1995 and 2015 (Fig. 7). The probability density curves of BMI Z-scores presented a clear right-shift trend over time in children aged 3- < 7 between 1985 and 2015 (Fig. 8).

#### Wasted

**Fig. 9:**
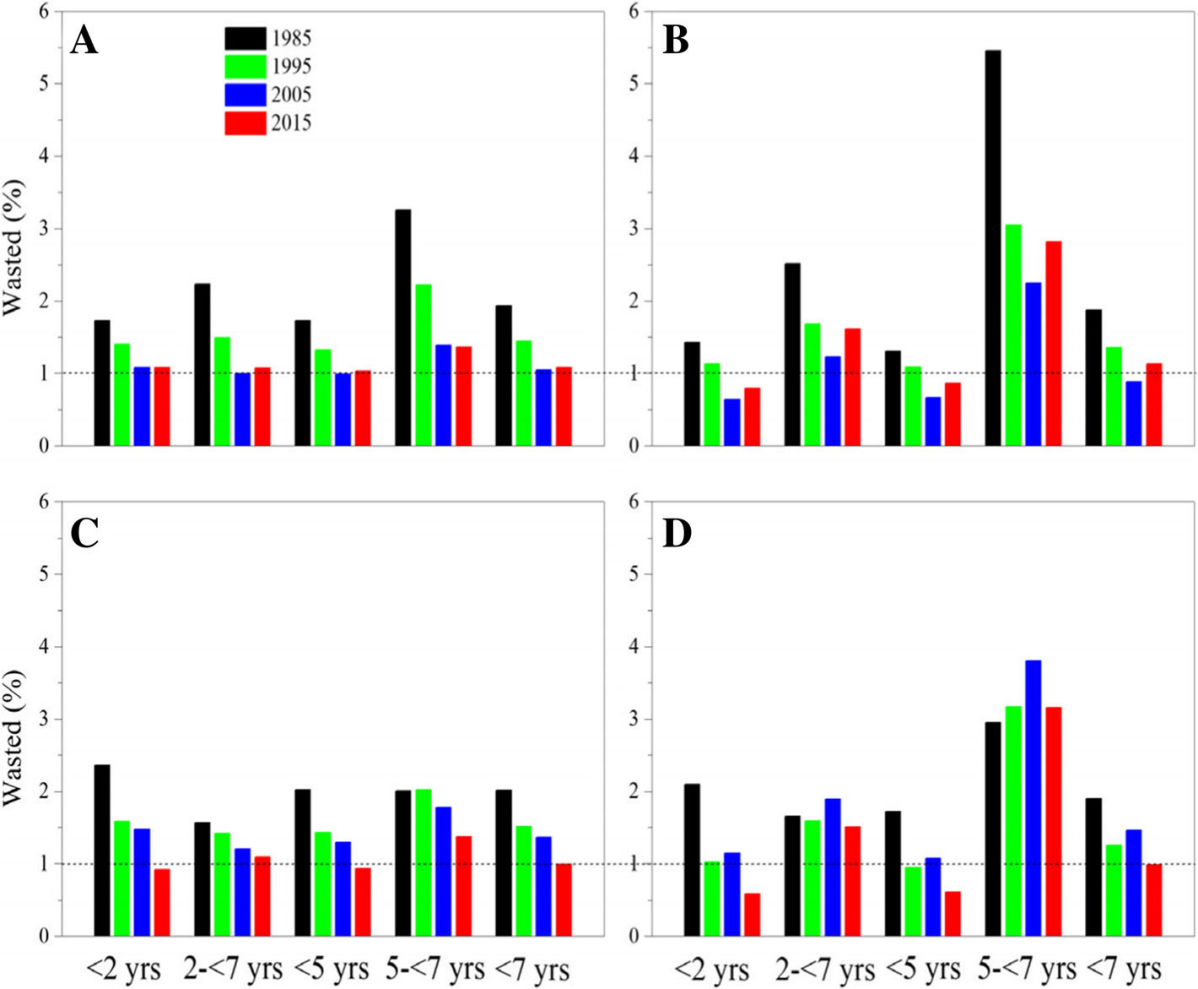
Thirty-year trends in wasted prevalence in children by age, sex and area, 1985–2015. (**a**: urban boys, **b**: urban girls, **c**: suburban boys, **d**: suburban girls)

The wasted prevalence dropped from 1.68 to 0.87% for children under 5 years and from 3.41 to 2.17% for children aged 5- < 7 from 1985 to 2015 (Table 3). By 2015, the prevalence under 5 years dropped to 1.03% for urban boys, 0.86% for urban girls, 0.94% for suburban boys and 0.62% for suburban girls, but the prevalence aged 5- < 7 lingered about 1.36, 2.82, 1.37 and 3.16% respectively (Fig. 9).

#### Overweight and obesity

**Fig. 10:**
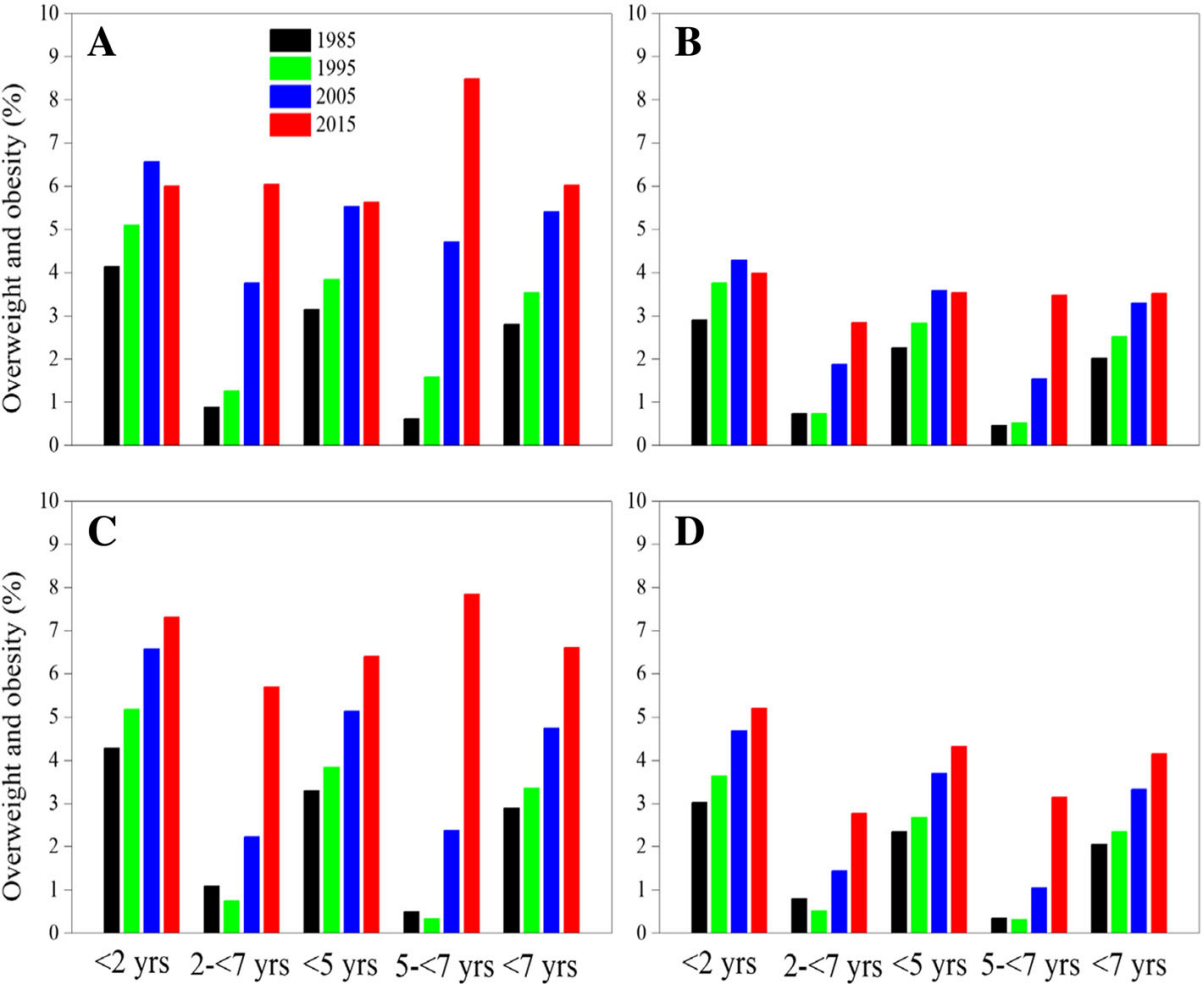
Thirty-year trends in overweight and obesity prevalence in children by age, sex and area, 1985–2015. (**a**: urban boys, **b**: urban girls, **c**: suburban boys, **d**: suburban girls)

The possible risk of overweight, overweight and obesity prevalence increased from 7.38 to 16.91%, the overweight and obesity from 0.87 to 4.34% and the obesity from 0.17 to 0.86% for children aged 2- < 7 from 1985 to 2015 (Table 3). The overweight and obesity prevalence almost did not increase between 1985 and 1995 and increased remarkably from 1995 to 2015 (Fig. 10). The increasing rates of overweight and obesity prevalence in suburban children first outnumbered those in urban children aged 2- < 7 during 2005–2015, with suburban boys 3.47% versus urban boys 2.29%, and suburban girls 1.33% versus urban girls 0.96%.

#### Overweight overtaking wasted or underweight in 2005 and onward

Between 1985 and 2005 the overweight (including obesity) prevalence increased to 2.32% for children aged 2- < 7 and during this period the wasted declined to 1.32% and the underweight decreased to 1.27% (Table 3). The overweight prevalence overtook the wasted or underweight in children aged 2- < 7 in 2005 and onward.

## Discussion

Chinese children are coming to a new stage of nutritional status with four distinct characteristics. First, the secular growth trend in Chinese children has entered a slow increasing phase. Second, the growth level of urban and suburban children has reached and surpassed the WHO 2006 growth standards. Third, under-nutrition is no longer main nutritional problem in Chinese cities. Fourth, over-nutrition increased remarkably over the past 20 years and the increasing rate of the prevalence in suburban children first outnumbered urban children in recent 10 years. The transition of nutritional status from under-nutrition to over-nutrition suggested child nutrition and health strategies should be adjusted and improved correspondingly as soon as possible.

The growth performance of urban and suburban children in nine cities has reached the level of well-nourished population. The socioeconomic positions of nine cities were at the forefront of their corresponding provinces or surrounding regions and those children in nine cities were considered to be born and raised in a relatively good condition of family income and parental education in China. Those children in nine cities had similar feeding patterns to developed countries [21]. The WHO 2006 growth standards represented the growth level of well-nourished population [22]. Our analysis for secular trends confirmed the growth level of children in nine cities experienced the process that lowered the WHO 2006 growth standards in 1995 and before, reached in 2005 and surpassed in 2015. Chinese Nutrition and Health Surveillance during 2010–2013 also showed the growth level of children under 6 years was higher than the WHO 2006 growth standards [23]. The prevalence of under-nutrition including stunting, underweight and wasted in nine cities at each survey year was lower than global average over the same period [24, 25].

Under-nutrition is no longer main nutritional problem in Chinese cities. According to our monitoring results, the stunting, underweight and wasted prevalence in children under 5 years continued to decline in nine cities over the past 30 years and descended to less than 1% of the level by 2015. Data from Chinese Nutrition and Health Surveillance also showed that China has made encouraging progress in nutritional status of children in recent years [26, 27]. Due to the regional inequality of socioeconomic status in the vast territory of China, under-nutrition still posed a big threat to the health of children in poor rural areas [27, 28]. Children in large cities in contrary to poor rural areas are facing the threat of over-nutrition [29].

Over-nutrition has become a serious public health problem in Chinese cities. Children obesity continued to increase rapidly in urban, suburban and rural areas over the past decades and faster increasing rates were observed in suburban and rural areas in recent years. Our data illustrated the increasing rates of overweight and obesity prevalence were faster in suburban children than urban children during 2005–2015, with suburban boys 3.47% versus urban boys 2.29% and suburban girls 1.33% versus urban girls 0.96% aged 2- < 7 years. Data from Chinese National Survey on Students’ Constitution and Health illustrated that during 2005–2010 the annual increase rates of obesity prevalence among 7- < 18-year students were rural boys 0.34% versus urban boys 0.30%, and rural girls 0.17% versus urban girls 0.10% [30] and during 2010–2014 the annual increase rates among these four groups were 0.79% versus 0.62, and 0.49% versus 0.42% [31]. Data from Chinese Nutrition and Health Surveillance also manifested the increasing rates of obesity prevalence in 7- < 18-year rural children (3.6%) outstripped urban children (3.1%) during 2002–2012 [32]. A large-scale longitudinal, household-based survey in China implied that overweight and obesity increase in Chinese children was associated with socioeconomic status, nutrition transition and sedentary behaviors [33, 34].

We observed two interesting phenomena which may be valuable to adjust swiftly and deliberately child nutrition and health strategies from primarily reducing under-nutrition prevalence to controlling rapid weight gain and promoting integrated early development. First, the height trend presented a “turning point” from rapid to slow in recent 10 years. Height and GDP were positively correlated at the national level [35]. Economic upturn promoted a positive height trend and economic downturn suggested a reduction in the speed of positive trend [35, 36]. China’s economy has been growing at a high speed since the 1978 economic reform, but the effect of GDP per capita on height trend seemed to be a little weaker between 1985 and 2015 than between 1985 and 2005. Based on the correlation coefficient of height trend and GDP per capita we inferred that height trend may be strongly associated with GDP per capita only at a certain stage of economic development, suggesting children in Chinese cities were close to their genetic growth potential at the population level. Second, the “time point” of overweight prevalence overtaking the wasted or underweight emerged in 2005. Under-nutrition decreased sharply from 1985 to 2005 and declined slightly from 2005 to 2015, but over-nutrition increased remarkably in recent 10 years and the increasing rate was faster in suburban children than urban children, suggesting China has been joining the fatter nations. Chinese school-aged children aged 7- < 18 years have also shifted from problems of under-nutrition to over-nutrition in recent 10 years [37]. If over-nutrition in childhood was not effectively controlled, it would affect the future population quality and bring heavy disease burden.

### Limitations

Several potential limitations should be noted. First, the NSPGDC only covered urban and suburban areas in China (not rural areas), the survey data were not sufficiently representative of rural areas in China where the growth performance and nutritional status may be not exactly the same as the results of this study. Owing to the regional inequality of socioeconomic status, China faced the double burden of over-nutrition and under-nutrition/nutrient deficiency among children [38]. Second, the NSPGDC excluded those children with obvious unhealthy height and weight (pathological obesity and short stature) which may lead to a slight underestimation for the prevalence. Third, the sample sizes contributing to the association analysis were actually four, the distributions of height Z-score and GDP per capita and IMR did not meet normal distributions, and theoretically it may be more proper to use Spearman rank correlation than Pearson correlation, but Spearman correlation could not differentiate the change of the coefficients (equal to 1 for height Z-score with GDP and − 1 with IMR for all the subgroups) in the condition of slowing height trend in 2015 due to the identical or reverse identical ranks for the aforementioned three variables. Therefore, we eventually used Pearson correlation to express the intensity of the associations.

## Conclusions

Progress and challenges from China may be helpful to further improve child nutrition and health strategies for many parts of the world which are undergoing transitions from low- to middle-income. China has achieved a sharp decline of stunting, underweight and wasted prevalence over the past 30 years, which suggested the sustainable socioeconomic development including but not limited to the improvement of living standards, medical and health conditions and education level were repeatable and reliable experience for promoting children’s nutritional status of a population. Our findings further suggested the window period of adjusting child nutrition and health strategies from reducing under-nutrition prevalence to controlling over-nutrition epidemic would be emerging when a population presented a slowing secular growth trend and its overweight prevalence overtook the wasted or underweight. China is facing the threat of over-nutrition and controlling overweight and obesity is a new mission in China in today and future. For China and some emerging countries or regions that are undergoing the transition from under-nutrition to over-nutrition, child nutrition and health strategies should be both realistic for helping children achieve their genetic growth potential and reducing under-nutrition prevalence and forward-looking for controlling rapid weight gain and promoting integrated early development.

## Abbreviations

BMI: Body mass index
GDP: Gross domestic product
IMR: Infant mortality rate
NSPGDC: National Survey on Physical Growth and Development of Children
SD: Standard deviations
WHO: World Health Organization
